# Prevalence of rheumatic heart disease in Zambian school children

**DOI:** 10.1186/s12872-018-0871-8

**Published:** 2018-07-03

**Authors:** Sherri Schwaninger, John Musuku, Mark E. Engel, Patrick Musonda, Joyce Chipili Lungu, Elizabeth Machila, Agnes Mtaja, Evans Mulendele, Dorothy Kavindele, Jonathan Spector, Brigitta Tadmor, Marcelo M. Gutierrez, Joris Van Dam, Laurence Colin, Aidan Long, Mark C. Fishman, Bongani M. Mayosi, Liesl J. Zühlke

**Affiliations:** 1 Novartis Institutes for BioMedical Research, Inc., 220 Massachusetts Avenue, 542B, Cambridge, MA 02139 USA; 2 Department of Paediatrics & Child Health, University Teaching Hospital, Lusaka, Zambia; 3 Department of Medicine, Groote Schuur Hospital and University of Cape Town, Cape Town, South Africa; 4 School of Public Health University of Zambia, Lusaka, Zambia; 5 Global Health, Novartis Institutes for BioMedical Research, Cambridge, MA USA; 6 Novartis Institutes for BioMedical Research, Cambridge, MA USA; 7 Global Health, Novartis Institutes for BioMedical Research, Basel, Switzerland; 8 Global Drug Development, Novartis Institutes for BioMedical Research, Cambridge, MA USA; 9 Department of Allergy and Clinical Immunology, Massachusetts General Hospital, Boston, MA USA; 10 Harvard Department of Stem Cell and Regenerative Biology, Harvard University, Cambridge, MA USA; 11 Department of Paediatrics, Red Cross War Memorials Children’s Hospital, Cape Town, South Africa; 12 Department of Medicine, University of Cape Town, Cape Town, South Africa

**Keywords:** Rheumatic heart disease, Rheumatic fever, Screening, Cardiovascular disease, Control strategies, Epidemiology, Echocardiography, Zambia, Prevalence, Streptococcal pharyngitis

## Abstract

**Background:**

The large global burden of rheumatic heart disease (RHD) has come to light in recent years following robust epidemiologic studies. As an operational research component of a broad program aimed at primary and secondary prevention of RHD, we sought to determine the current prevalence of RHD in the country’s capital, Lusaka, using a modern imaging-based screening methodology. In addition, we wished to evaluate the practicality of training local radiographers in echocardiography screening methods.

**Methods:**

Echocardiography was conducted on a random sample of students in 15 schools utilizing a previously validated, abbreviated screening protocol. Through a task-shifting scheme, and in the spirit of capacity-building to enhance local diagnostic and research skills, general radiographers based at Lusaka University Teaching Hospital (UTH) were newly trained to use portable echocardiography devices. Students deemed as screen-positive were referred for comprehensive echocardiography and clinical examination at UTH. Cardiac abnormalities were classified according to standard World Heart Federation criteria.

**Results:**

Of 1102 students that were consented and screened, 53 students were referred for confirmatory echocardiography. Three students had definite RHD, 10 had borderline RHD, 29 were normal, and 11 students were lost to follow-up. The rates of definite, borderline, and total RHD were 2.7 per 1000, 9.1 per 1000, and 11.8 per 1000, respectively. Anterior mitral valve leaflet thickening and chordal thickening were the most common morphological defects. The pairwise kappa test showed fair agreement between the local radiographers and an echocardiographer quality assurance specialist.

**Conclusion:**

The prevalence of asymptomatic RHD in urban communities in Zambia is within the range of results reported in other sub-Saharan African countries using the WHF criteria. Task-shifting local radiographers to conduct echocardiography was feasible. The results of this study will be used to inform ongoing efforts in Zambia to control and eventually eliminate RHD.

**Trial registration:**

The study was registered on clinicaltrials.gov (#NCT02661763).

## Background

Rheumatic heart disease (RHD) is a common and neglected health problem in the developing world and in indigenous regions of some high-income countries [1, 2]. Greater than 33 million people have RHD and nearly 320,000 die from the disease each year [3–5]. In sub-Saharan Africa, studies from multiple countries report that approximately 0.5–3% of school-age children have echocardiographic signs of definite or borderline disease according to World Heart Federation (WHF) criteria [6–9].

RHD is a preventable sequela of streptococcal pharyngitis, commonly known as “strep throat.” A small percentage of patients with untreated streptococcal pharyngitis will develop autoimmune-mediated acute rheumatic fever (ARF) and then RHD, which is characterized by heart valve damage and progressive cardiac failure [10]. No specific treatment for RHD exists but, if detected early, continuous antibiotic prophylaxis (also known as “secondary prevention” of RHD) can decrease ARF attack rates and mitigate progression of heart disease [11–13]. Many patients will eventually require complex pharmaceutical regimens and surgical intervention [14]. Too often, RHD leads to chronic morbidity and premature death [15].

RHD has been poorly addressed in Zambia. Prevalence was estimated at 1.3% when measured nearly three decades ago by auscultation [16]. In 2001, a review of 200 case notes for pediatric cardiac patients at Lusaka University Teaching Hospital (UTH), Zambia’s main referral hospital, revealed that 70% were admitted for RHD (J Musuku, unpublished data). Currently, UTH provides services for approximately ten RHD outpatients each week and on average five inpatients daily. There are no specialized services for RHD available in the country outside of Lusaka.

After receiving specialty training in paediatric cardiology in South Africa, a UTH-based pediatrician mobilized academic colleagues, the Ministry of Health, local professional societies, and a pharmaceutical company in a multi-faceted effort to eliminate RHD in Zambia [17, 18]. Priorities of this program were structured according to the “A.S.A.P.” framework (Awareness-raising, Surveillance, Advocacy, and Prevention) established by the Pan-African Society of Cardiology as a strategy to reduce morbidity and mortality associated with RHD in sub-Saharan Africa [19, 20].

As part of this effort, the prevalence of RHD was measured in Zambian school children using handheld echocardiography [21–23] and a recently validated simplified screening protocol based on the WHF criteria for echocardiographic diagnosis of RHD [24, 25]. In order to assure effective follow-up care for students identified to have RHD, selected health clinics in Lusaka were enrolled into a novel RHD control program that consisted of training, ongoing supportive supervision, and maintenance of adequate medicine stores [26, 27].

The opportunity of this study was also used to build local echocardiographic capabilities that could be applied to subsequent clinical and research activities in Zambia [28]. Data from this investigation were anticipated to inform health programs combating RHD and to support advocacy for RHD patients.

## Methods

### Study design and oversight

This was an observational cross-sectional study designed to measure the prevalence of RHD in school children in Lusaka, the capital of Zambia. The study was registered on clinicaltrials.gov (#NCT02661763) and approved by the Biomedical Research Ethics Committee at the University of Zambia, the Ministry of Health, the Ministry of Education, and the Ministry of Community Development, Mother and Child Health. Data collection took place between September and December in 2015.

### Study population

The study population was drawn from a random sample of children in grades seven through twelve attending basic, secondary, and high schools in Lusaka. Screening was focused on older school children (rather than younger school children) based on evidence that they are at higher risk of RHD [9, 29, 30].

Written informed consent for participation in the study was required from parents or legal guardians. Information about the study and consent forms were sent home with students several weeks before each screening session. Consent forms were written in English, Nyanja and Bemba, which are the most common languages in Lusaka. Children were also verbally informed about the study at the time of screening and were required to provide written assent with their name or thumbprint in order to participate.

Inclusion criteria were all children whose parents consented, who provided assent, and who were present at the time the screening was conducted. Exclusion criteria included conditions that precluded the performance of transthoracic echocardiography (e.g., skin infection of the chest).

A member of the study team approached the administrative head of each selected school to invite their school to participate. Awareness messages (flyers and posters) that described the study were distributed, and study staff briefed teachers whose classrooms were to be involved. The classrooms selected to participate in each school were determined at random and approximately 60–80 students were enrolled from each school.

### Study procedures

Prior to screening, each participant’s parent or guardian completed a questionnaire that collected information on demographics and the participant’s general health history. On the day of screening, the study team (consisting of a field coordinator, pediatric nurses, radiographers, data entry staff, and sometimes a physician) organized a private screening area and ensured that a member of the school staff would be present during interactions between study staff and participants.

**Fig. 1:**
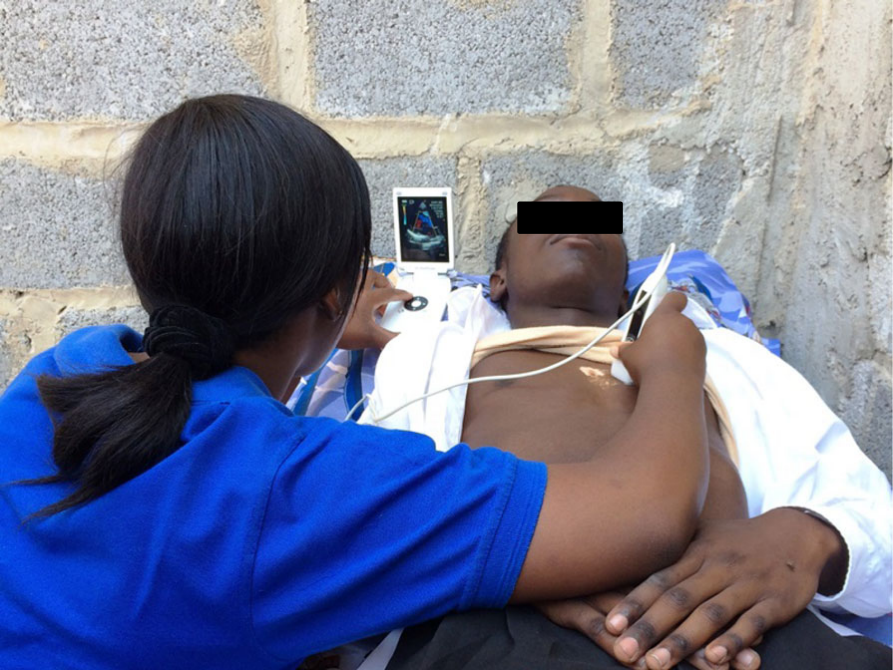
A radiographer from Lusaka University Teaching Hospital uses ultraportable echocardiography to screen an adolescent for rheumatic heart disease as part of the study (photograph used with permission of participant)

Study staff conducted a focused history and physical examination with each participant, specifically to assess for pharyingitis, RF, or RHD. Study staff then performed a screening echocardiogram using the abbreviated screening protocol and a handheld ultrasound device (Fig. 1). The overall process generally lasted 15–20 min for each participant; the echocardiographic screening component lasted about 5 min per participant. Refreshments were provided after the screening. The study staff prioritized flexibility in scheduling so that interruption of regular school activities was minimized.

### “eRegister” database

Data obtained at the time of screening was entered through mobile electronic devices (Samsung Galaxy 4 Tab computer tablets) into a digital system (“eRegister”) that was developed specifically for the study [31]. The system used a well-established e-health platform (“CommCare” by Dimagi) [32, 33], which includes a customizable mobile health data collection application and an online portal for secure data viewing and reporting. All access to the digital platform including mobile submissions was done over HTTPS (hypertext transfer protocol secure) and was cryptographically secure. Mobile devices were password-locked. Echocardiograms were also saved in the system; images and videos were temporarily stored on the hard drive of the ultrasound device and then transferred to the eRegister.

### Echocardiographer training

Six general radiographers from UTH were recruited to participate in the school screening study, and three ultimately completed training and remained part of the study team throughout the study period. Training comprised classroom-style and hands-on workshops involving actual RHD patients at UTH and supportive supervision during school screening [28]. Training activities took place periodically over 12 months beginning May 2014. Training was structured to accommodate the practical considerations of trainees, who continued to work as general radiographers at UTH.

Radiographers were trained to use a handheld machine (General Electric V-Scan, General Electric Company, Fairfield, CT, USA) and to screen participants for evidence of RHD using a recently validated, simplified protocol called FOCUS (an acronym for FOCused method Utilizing hand-held echocardiography in Screening for RHD) [24]. This protocol incorporates key aspects of the much more complex (and definitive) WHF protocol for echocardiography-based RHD diagnosis.

The abbreviated protocol utilizes a single view (the parasternal long-axis view) and a single measurement (regurgitation jet in the mitral valve, which is implicated in most RHD cases). According to the protocol, the mitral valve is interrogated, first without and then with color Doppler. A regurgitation jet-length greater or equal to 2 cm (measured from the vena contracta to the last pixel of the regurgitant jet using the V-Scan radius measuring tool) constitutes a positive result.

Following the first intensive training of radiographers that started in May 2014, school screening initially began in October 2014. However, quality assurance checks by study investigators (JM, LZ) revealed suboptimal performance by the newly trained radiographers. The study was put on hold pending additional training and practice. The study re-started in September 2015 after the radiographers’ proficiency was confirmed through quality assurance checks by a blinded external expert (described below). Improvement in radiographers’ performance was attributed to repeated practice, real-time guidance during periodic visits by volunteer echocardiography technicians from abroad, and training with the assistance of RHD patients at UTH.

When the study re-started, two mechanisms were implemented to help ensure successful screening: (1) four international echocardiography technicians spent a total of 8 weeks accompanying study staff during school screening to support the work of local radiographers; and (2) all screening echocardiograms were reviewed remotely by a professional third-party echocardiographer (VirtualScopics, University of Rochester, USA) who accessed de-identified scans through the eRegister and conducted independent review of the echocardiograms recorded by the field team.

### Screening and confirmatory echocardiography

Echocardiographic screening was performed using V-Scan handheld machines. If a participant screened positive at the school or through the VirtualScopics quality assurance (VSQA) review, study staff informed the participant’s parents or guardians and arranged follow-up at UTH where a member of the study team (JM) performed comprehensive echocardiography using a standard portable ultrasound machine (Philips CX 50 or Sonosite M-Turbo).

The follow-up echocardiogram was conducted according to evidence-based consensus WHF guidelines [25] using 2D, continuous-wave, and color-Doppler to assess for morphological abnormalities or pathological mitral or aortic valve regurgitation. The echocardiograms of participants that underwent follow-up echocardiography were classified as “normal,” “definite RHD,” or “borderline RHD.”

As described by WHF, definite RHD corresponds to echocardiographic changes that are likely to be rheumatic in origin assuming the clinical context is consistent. Borderline RHD corresponds to echocardiographic changes that may represent early RHD in individuals aged 20 years or less. The borderline category was established by WHF because young people may benefit from early detection of RHD and initiation of antibiotic prophylaxis [25]; progression of heart disease in patients with borderline RHD has been documented [34, 35].

### Student disposition

**Fig. 2:**
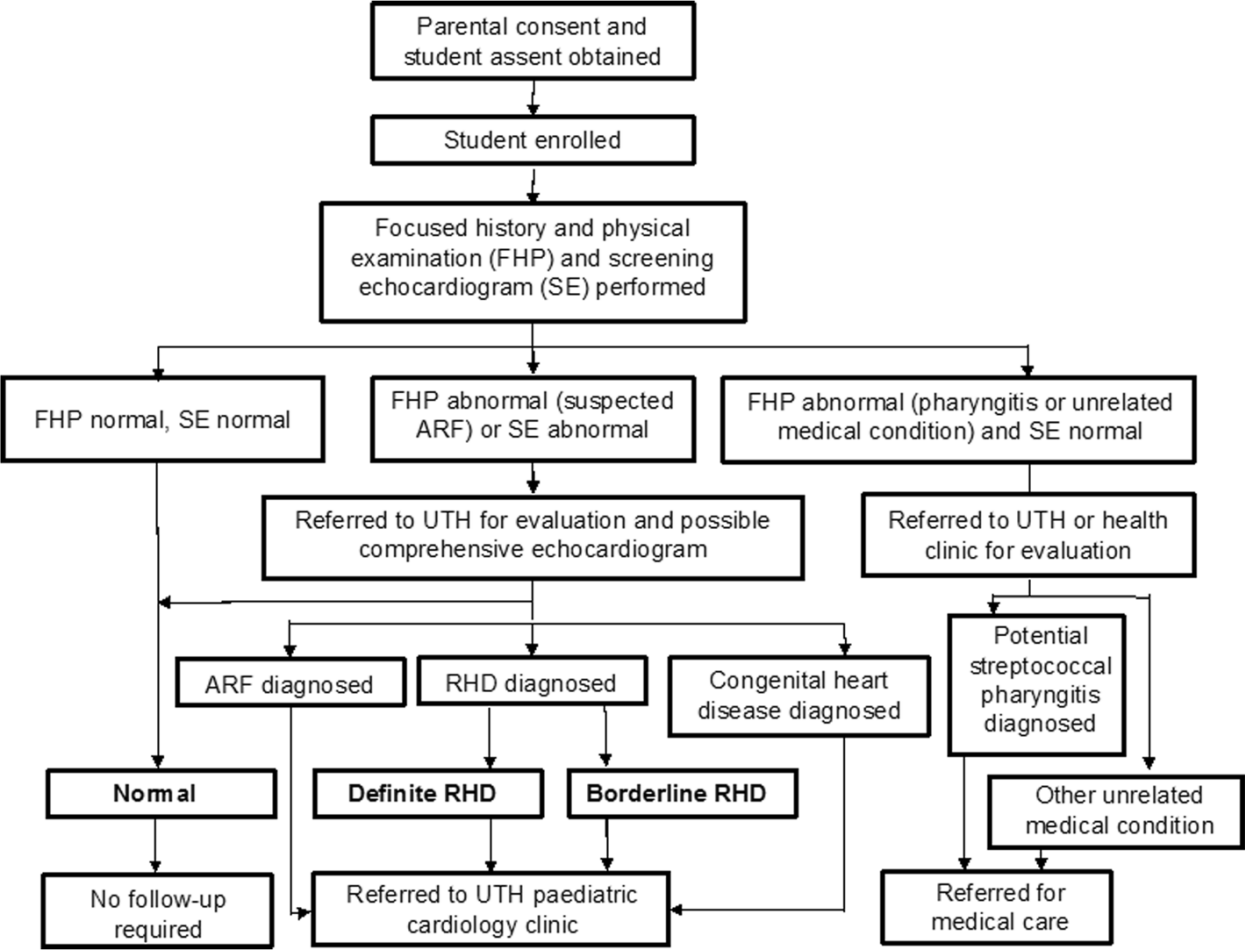
Student disposition in study methodology

Participant disposition was determined by the results of school screening and confirmatory echocardiography (Fig. 2). No follow-up was required if the focused history and physical examination, the screening echocardiogram, and the VSQA review were normal.

When the physical examination revealed pharyngitis and the screening echocardiogram/VSQA review was normal, the participant was referred to UTH or a local health clinic for further evaluation and management, including primary prevention of RHD. When the screening echocardiogram revealed any abnormality, or there was a clinical suspicion of ARF, the participant was referred to UTH for a comprehensive evaluation and quantitative echocardiogram in order to assess for ARF or RHD. Participants with definite or borderline RHD were referred to the pediatric cardiology clinic at UTH or a local health clinic for long-term follow-up including antibiotic prophylaxis and repeat echocardiography on a 6- or 12-monthly basis [14, 34].

Participants with cardiac abnormalities due to congenital heart disease or other non-rheumatic problems (for example, skin or dental problems) were referred for follow-up evaluation and treatment at UTH or their local clinic in accordance with standard medical care in Zambia.

### RHD control program in health clinics

Participants that were identified to have borderline or definite RHD were referred for follow-up management which included the recommendation for antibiotic prophylaxis (with oral penicillin or monthly injections of benzathine penicillin) according to national guidelines. Penicillin-allergic individuals were alternatively offered erythromycin. UTH was an inconvenient location for some participants to routinely travel for follow-up and therefore selected health clinics in Lusaka that were located near the study schools were enrolled in an RHD control program [27].

As part of this program, study nurses and physicians conducted an introductory on-site training workshop, disseminated educational materials to staff and patients, and performed monthly or bi-monthly supportive supervisory visits. The training taught knowledge and skills to support effective primary prevention (i.e., treatment of bacterial pharyngitis with a single dose of benzathine penicillin to decrease risk of RF), secondary prevention (i.e., continuous antibiotic prophylaxis in children with RHD to mitigate progression of heart disease), and pharmacovigilance. Details of the training are described previously [26]. Reliable availability of high-quality benzathine penicillin at UTH and participating clinics in Lusaka was faciliated by a product grant to the Ministry of Health by Sandoz, the generics division of Novartis.

### Sample size considerations

The total school population in Lusaka within which the study took place is approximately 500,000. Since previous studies demonstrated that RHD prevalence increases in older children [9, 29, 30], this study focused on prevalence in children attending grades 7 and above. A minimum sample size of 1024 participants in 15 randomly selected schools was calculated based on precision around a point estimate of 1% RHD prevalence in Lusaka. This was felt to be a conservative estimate in light of similar studies from other countries in sub-Saharan Africa [8, 29, 30, 36]. The sample size also accounted for a presumed rejection rate of 9%. With this sample, it was determined that the prevalence of RHD could be estimated within 0.7 percentage points of precision with 95% confidence. A 1.2 design effect due to clustering in the sampling design was assumed given that some participants were enrolled from within clusters (i.e., participants enrolled from the same classrooms and schools were more likely to be similar in unintended ways relative to a completely random population).

Variation in the total number of eligible individuals in each school was expected, as was the total size of each school. We accounted for this variation in the sampling process by weighting the primary sampling unit (the school) and selecting according to size, using probability proportional to size (PPS) sampling [37]. The sampling weights were also used in the analysis to control for possible misspecifications of standard errors in each cluster.

As discussed above, the study was temporarily halted early on to allow for improved training of radiographers. A formal assessment of radiographers’ performance using the screening protocol was then conducted by VSQA review, and schools were re-randomized prior to re-starting the study. Fifteen schools included in the study were randomly selected from a Ministry of Education database of all 119 basic, secondary, and high schools in Lusaka. Within each randomly selected school, one grade was randomly selected (grades 7–12). Multiple classrooms in the selected grade were then randomly selected with the aim to enroll a minimum of 60 students per school. All students in the selected classrooms were eligible to participate if consent and assent were completed.

### Analysis plan

The study measured prevalence expressed as a proportion per 1000 individuals using standard statistical analysis tools. The estimate of the standard error accounted for clustering effects and was considered in the confidence interval computation. Sub-analyses using a similar approach were conducted by age, gender, and selected additional demographic parameters, though it was not expected that these variables would have adequate power for precise estimates of prevalence for these subgroups. Categorical data were compared by chi-squared analyses, chi-squared for trend, or expressed as risk ratios with 95% confidence intervals as appropriate. To assess the reliability of the training program in skills-building for the local radiographers, a pairwise kappa test was performed.

## Results

**Fig. 3:**
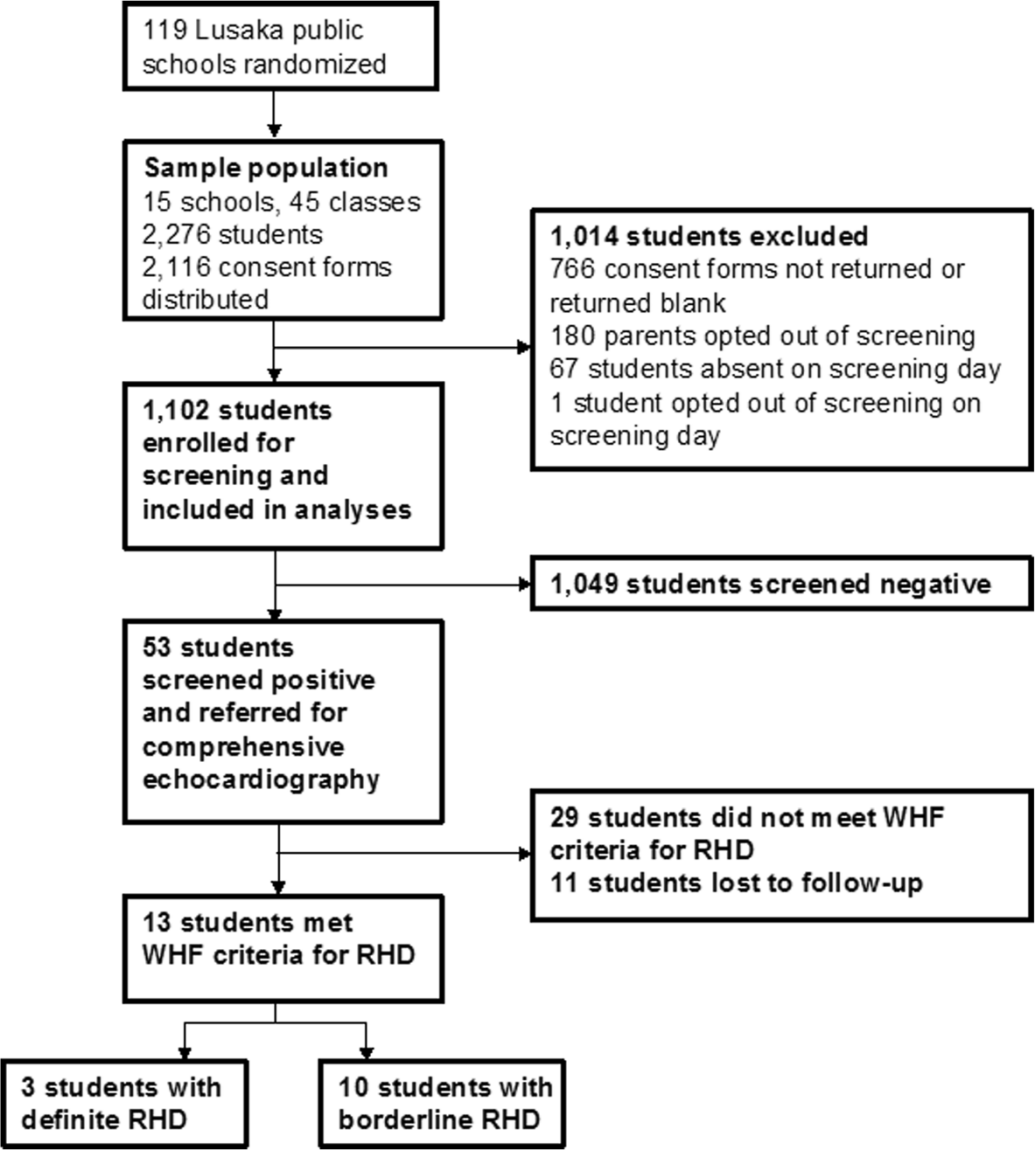
Flowchart of schools of students involved in the study

The sample population consisted of 2276 students in 15 schools that were eligible for participation. Consent was attempted for 2116 students that were in attendance in school on the day that consent forms were distributed (160 students were absent on that day). A total of 1102 students ultimately advanced through the consent process and underwent echocardiographic screening as part of the study (Fig. 3).

Sociodemographic characteristics of students included in the study revealed a slight predominance of female participants, and a majority of students from lower socioeconomic strata as determined by the neighborhood in which they lived (Table 1).

**Table 1:** Sociodemographic characteristics of 1102 students screened for RHD in Lusaka, Zambia

Characteristic	n(%)
Gender	
Female	590 (54)
Male	512 (46)
Age (years)	
Mean (SD)	15.4 (1.9)
Median	15
10–12	48 (4.4)
13–15	506 (45.9)
16–18	443 (40.2)
19–21	40 (3.6)
22–24	2 (0.2)
Unobserved	63 (5.7)
Socioeconomic status	
Lower	658 (59.7)
Higher	330 (29.9)
Unobserved	114 (10.3)
Schools	
Basic schools	
Yotam Muleya	102 (9.3)
Namando	86 (7.8)
State Lodge A	62 (5.6)
Hillside	71 (6.4)
Kaunda Square	74 (6.7)
Kalingalinga	21 (1.9)
Lotus	75 (6.8)
Twashuka	52 (4.7)
Kamulanga	86 (7.8)
Secondary schools	
Matero	123 (11.2)
Nyumba Yanga	100 (9.1)
High schools	
St. Patrick Girls	91 (8.3)
Kamulanga	36 (3.3)
Munali Boys	51 (4.6)
Kabulonga Boys	72 (6.5)

Most (86%) students were aged 13–18 years. Ten students were identified by study nurse-coordinators to have pharyngitis at the time of screening and were referred for medical follow-up. Fifteen students were found to have other medical problems during screening (including dental caries and rashes) and were also referred for medical evaluation. One student was found by screening echocardiography to have congenital heart disease.

Fifty-three students screened positive for RHD by portable echocardiography at schools and were referred for comprehensive echocardiography at UTH. Of those, 42 students followed-up and received confirmatory echocardiography. There were no gender differences in the cohort of children that screened positive. On confirmatory echocardiography, it was found that 3 students met WHF criteria for definite RHD, 10 students met criteria for borderline RHD, and 29 students did not meet criteria for any category of RHD. The confirmed prevalences of definite, borderline, and total RHD were therefore 2.7 per 1000 (95% CI 0.6–8.0), 9.1 per 1000 (95% CI 3.8–15.6), and 11.8 per 1000 (95% CI 5.7–15.6), respectively. Despite extensive and prolonged efforts by the study coordinators to follow-up students that screened positive, eleven students were lost to follow-up between the time they screened positive at schools and the time of a comprehensive echocardiographic assessment at UTH.

Demographic and clinical characteristics of the students that were diagnosed with definite and borderline RHD are shown in Table 2.

**Table 2:** Demographic and clinical characteristics of 13 students identified to have definite and borderline RHD

Characteristic	n (%)
Gender	
Female	8 (62)
Male	5 (38)
Age (years)	
Mean (SD)	15.3 (1.9)
Median	16
Range	13–18
Socioeconomic status	
Lower	10 (77)
Higher	1 (8)
Unobserved	2 (15)
Definite RHD criteria met [25]	
Pathological MR and at least two morphological features of RHD of the MV	3
MS mean gradient ≥ 4 mmHg	0
Pathological AR and at least two morphological features of RHD of the AV	0
Borderline disease of both the AV and MV	0
Borderline RHD criteria met [25]	
At least two morphological features of RHD of the MV without pathological MR or MS	0
Pathological MR	10
Pathological AR	0

Acronyms, *AR* atrial regurgitation, *AV* atrial valve, *MR* mitral regurgitation, *MS* mitral stenosis, *MV* mitral valve, *SD* standard deviation

Each of these students had abnormalities of the mitral valve, with the most commonly observed abnormal morphological features being anterior mitral valve leaflet thickening and chordal thickening. The aortic valve was not affected in this group of students.

Prior to the main study initiation a formal assessment of the capabilities of the local radiographers to successfully implement the abridged echocardiographic screening protocol was conducted. The echocardiograms of 471 students screened by the local radiographers were reviewed by the VirtualScopics quality assurance (VSQA) specialist. The pairwise kappa test showed fair agreement between the local radiographers and the VSQA quality assurance radiographer (agreement, 97%; ϰ = 0.54; SE = 0.11). The results are listed in Table 3.

**Table 3:** Measurement of performance of local radiographers in echocardiography-based screening (“negative” refers to a normal screening evaluation; “positive” refers to a screening evaluation that is abnormal)

Number of screening tests	Local radiographers	Quality assurance specialist
450	Negative	Negative
12	Negative	Positive
8	Positive	Positive
8	Positive	Negative

Of the 53 students referred for comprehensive echocardiography at UTH, 24 had screened negative at the school screening but the VSQA review indicated mitral regurgitation with jet length ≥ 2 cm in at least one view. From this cohort, 18 students followed-up with a confirmatory echocardiogram resulting in the diagnosis of one definite RHD and five borderline RHD cases. There was concordance between the local radiographers and the VSQA review for 28 students that screened positive, and 23 of these students followed-up with a confirmatory echocardiogram that resulted in the diagnosis of two definite RHD and five borderline RHD cases. One student screened positive at school and the VSQA review was negative; the confirmatory echo diagnosis was normal.

## Discussion

This study found a RHD prevalence of 11.8 per 1000 in Zambian school children. In addition, this study demonstrated the practicality of successfully training radiographers in echocardiography-based screening. The main finding that 1.2% of school children have evidence of borderline or definite RHD is consistent with investigations using similar methodologies in urban settings of other sub-Saharan African countries (Table 4).

**Table 4:** Summary of RHD prevalence studies using echocardiography conducted in sub-Saharan Africa. More than 27,000 children have been screened for RHD using echocardiography in this region. The variability in findings of RHD prevalence between countries is not fully understood

Year reported	Country (location)	Population studied	Methodology	RHD prevalence
1996	Kenya (Eldoret) [49]	1150 school children in grades 1–8 in two schools	Colour-flow echocardiography and a portable generator using investigator-defined criteria	Definite 0.3%
2007	Mozambique (Maputo) [30]	2170 randomly selected children aged 6–17 years in six schools	Portable echocardiography at schools using investigator-defined criteria	3%
2012	Uganda (Kampala) [29]	4869 children aged 5–16 years in six randomly selected schools	Portable echocardiography at schools using 2006 WHO/NIH criteria	Possible 1%; probable 0.4%; definite 0.2%
2014	Eritrea (multiple cities) [50]	684 adolescents aged 13–24 randomly selected from nine schools	Portable echocardiography at schools using investigator-defined criteria	Suspected 3.3%; definite 4%
2015	Senegal (Dakar) [36]	2019 children aged 5–18 years in 16 schools	Portable echocardiography at schools using WHF criteria	Borderline 1.1%; definite 0.05%
2015	Ethiopia (Jimma) [8]	2000 children aged 4–24 years from randomly selected schools	Echocardiography at a university-affiliated field research center using WHF criteria	Borderline 1.4%; definite 1.7%
2015	South Africa (Cape Town) [8]	2720 children aged 4–24 years in randomly selected schools	Portable echocardiography in a mobile clinic using WHF criteria	Borderline 1.5%; definite 0.5%
2015	South Africa (Free State and Northern Cape) [51]	1015 children aged 14–22 years in grades 10–12	Portable echocardiography at schools using investigator-defined criteria	Definite 0.5%
2016	Malawi (Lilongwe) [7]	1450 children aged 5–16 years in three schools and surrounding communities	Portable echocardiography at schools and in surrounding communities using WHF criteria	Borderline 2.7%; definite 0.7%
2016	Uganda (Kampala) [52]	488 HIV-infected children aged 5–18 years from an outpatient clinic	Echocardiography at medical centers using WHF criteria	Borderline 0.4%; definite 0.4%
2016	Ethiopia (6 cities) [6]	3238 children aged 6–18 years in 28 randomly selected schools	Portable echocardiography at schools using WHF criteria	Borderline 0.5%; definite 1.4%
2017	Rwanda (Gasabo district) [53]	2501 children aged 6–16 years in 10 schools	Portable echocardiography at schools using WHF criteria	Borderline 0.5%; definite 0.2%

The finding is also similar to the single previous estimate of RHD in Zambia that was obtained several decades ago albeit that study used different screening (auscultation alone) and diagnostic criteria (not utilizing current World Heart Federation criteria) [16]. It is notable that this research did not take place in isolation; rather, from the start it was incorporated into a broader health systems strengthening program that included the majority of government health clinics in Lusaka and aims to provide tailored support for the prevention and treatment of RHD in Zambia.

We trained radiography technicians that had little or no previous experience with echocardiography to perform school-based screening. This was necessary because health workers in Lusaka are not skilled in cardiac echocardiography except for a few health professionals at UTH. The feasibility of task-shifting simple echocardiographic screening protocols to non-expert health workers has been demonstrated in similar settings [38–41]. In Zambia, this approach necessitated greater resources to conduct training and assess their results, and it also delayed the study until the quality of the screening echocardiograms could be assured. However, the study team felt strongly about this approach as the investment in time and resources was viewed as a method for building local echocardiographic capabilities to support subsequent clinical and research activities in Zambia. Hence the screening for each student involved in the study was conducted by a local radiographer. Ultimately, the performance of the local radiographers in the study was characterized by significant inconsistencies when compared with the VSQA quality assurance review. Without the VSQA review, the school screening process would have missed nearly half of the students diagnosed with definite or borderline RHD. The challenges that were faced in providing local radiographers with the skills required to confidently and independently conduct screenings for RHD will be an important consideration in related future research in Zambia and may have implications for the design of similar research programs in other countries.

Various simplified echocardiography protocols have been utilized to facilitate population-based screening for asymptomatic RHD [42, 43], and these are now being used in conjunction with ultra-portable handheld echocardiography devices in an effort to further streamline the screening process [44, 45]. In this study we used the recently validated FOCUS screening protocol, which was previously shown in a controlled investigation to be 92% sensitive and 100% specific for definite RHD [24]. This protocol, which utilizes a single view and measurement, was particularly advantageous in the current study in which screening was conducted by general radiographers who lacked echocardiography experience. Of the 42 students that screened positive and underwent comprehensive follow-up echocardiography, 31% (*n* = 13) were diagnosed with definite or borderline RHD according to WHF criteria. We attribute the decreased specificity to “real-world” factors that include uncertainty with some measurements obtained in the “field” (i.e., at schools) and the motivation by first-time study radiographers and coordinators to be conservative in their screening assessments of children to minimize the risk of missing a student who may have RHD. The number of referred patients for confirmatory echocardiography did not impose an excessive burden on the study team.

Just over half of students that received a consent form and questionnaire were ultimately screened (Fig. 3). Of the consent forms that were distributed to school children to take home to their parents, 36% were not returned or were returned blank. Nine percent of families opted out of participation. The consent form included a questionnaire with 11 questions to obtain demographic information and the child’s health history, with a focus on the incidence and treatment of sore throat. We hypothesize that completing the questionnaire may have been perceived as burdensome to some parents/guardians. Also, students may have forgotten to give the form to their parents, or failed to bring a completed form back to school. Absenteeism was also a factor on screening day (5.7% of students).

A novel education and training program was, for the first time in Zambia, implemented in Lusaka clinics by the study team in order to assure appropriate follow-up clinical care for participants identified through the study to have RHD [26]. The program comprised an introductory on-site training workshop, dissemination of educational materials for staff and patients, ongoing supportive supervisory visits by UTH staff, and assessment of antibiotic stocks with replenishment of benzathine penicillin when necessary. Key elements of the program included guidance on when and how to administer secondary antibiotic prophylaxis, how to manage the unlikely event of penicillin allergy, and how to deliver primary prevention of RHD (i.e., detection and proper treatment of bacterial pharyngitis). At the time of the investigation eight clinics were enrolled that were selected specifically due to their geographic proximity to study schools. Since then the program has grown to include all 29 government clinics in the Lusaka municipality, and activities are underway to expand the RHD control program to other provinces beyond Lusaka. Future work will involve the exploration of innovative ways to scale up the RHD control program (such as the use of electronic training modules), and to provide broad access to educational materials through the Pan African Society of Cardiology (PASCAR).

Borderline RHD is an echocardiography-based diagnostic category established in recent years by the World Heart Federation to improve the sensitivity of early detection of RHD in individuals ≤ 20 years [25]. In theory, early diagnosis paves the way for clinical interventions that prevent or mitigate disease progression, although this has not been proven in practice [46]. Because this category is new and its natural history is not yet fully understood, a major research priority has been to determine optimal management of individuals with borderline RHD. In the current study participants with borderline RHD were offered antibiotic prophylaxis along with routine clinical follow-up and periodic echocardiography, an approach that was taken in some previously described programs [6, 8] and not others [7, 47]. A recent prospective observational study of 34 school children in South Africa diagnosed with borderline RHD were followed up after 5 years [48]; 16% continued to have borderline RHD and 16% progressed to definite RHD, which suggests that initiation of antibiotic prophylaxis in children with borderline disease may have clinical benefit if antibiotic prophylaxis prevents progression of latent RHD. A similar conclusion was drawn in a prospective cohort study of indigenous Australian children with borderline RHD who were found to be at increased risk of progression to definite RHD [34]. At this time the approach in Zambia is to initiate antibiotic prophylaxis in individuals with borderline RHD and to discontinue prophylaxis on a case-by-case basis according to each patient’s clinical evolution. We will refine these tactics as new data and recommendations from leading health authorities become available.

There were some limitations in this study. Eleven participants who screened positive for RHD at schools were lost to follow-up. In most cases, parents or guardians were successfully contacted but ultimately could not be persuaded to bring their children for a confirmatory echocardiogram at UTH. This was despite offers of travel reimbursement and numerous attempts over three months or more by study staff to facilitate follow-up, including repeated telephone calls and in-person visits back to schools to discuss with families and school staff. In many cases, appointments were made for follow-up visits but not kept. If future screening studies are conducted in Zambia, we aim to incorporate on-site confirmatory echocardiography such that definitive diagnoses can be made at the time of screening and obviate the need for subsequent visits. Another limitation of the study is that the population screened was limited to school-going children in the urban setting of Zambia’s capital; this group may not be representative of the entire country. The study was limited to Lusaka mainly because of resource considerations, including clinical staff that would be available to conduct the study. Access to healthcare is generally accepted to be greater in Lusaka than in rural and remote regions of the country, which may result in improved treatment of streptococcal pharyngitis and prevention of RF and RHD. Indeed, more patients with RHD are reportedly referred to UTH from communities that are located outside of Lusaka, although there is not yet a national patient registry or surveillance system in place. Finally, while the screening protocol was associated with a validated sensitivity above 90%, there remains the possibility that some participants with potential RHD erroneously screened negative which would have led to an underestimation of the disease prevalence in the population studied.

## Conclusions

The prevalence of RHD in Zambia is similar to that reported in neighboring countries and other low-resource settings globally. This information is being used to inform ongoing advocacy and education efforts designed for local health authorities, health workers, and the public. The abbreviated echocardiography screening protocol performed well in practice and may be considered for use in future prevalence studies. The conduct of this investigation was viewed as an opportunity to incorporate capacity-building for the local radiographers and to launch primary and secondary prevention programs in local clinics. The authors recommend prioritizing this approach whenever possible, even if it requires greater investment in time and other resources.

## Abbreviations

A.S.A.P.: Awareness-Raising, Surveillance, Advocacy and Prevention
AR: Atrial Regurgitation
ARF: Acute Rheumatic Fever
AV: Atrial Valve
CI: Confidence Interval
FOCUS: FOCused method Utilizing hand-held echocardiography in Screening for RHD
HTTPS: Hypertext Transfer Protocol Secure
MR: Mitral Regurgitation
MS: Mitral Stenosis
MV: Mitral Valve
PPS: Probability Proportional to Size sampling
RF: Rheumatic Fever
RHD: Rheumatic Heart Disease
SD: Standard Deviation
UTH: Lusaka University Teaching Hospital
VSQA: VirtualScopics quality assurance
WHF: World Heart Federation
